# The risk of metabolic syndrome after gestational diabetes mellitus – a hospital-based cohort study

**DOI:** 10.1186/s13098-015-0038-z

**Published:** 2015-05-12

**Authors:** Tiina Vilmi-Kerälä, Outi Palomäki, Merja Vainio, Jukka Uotila, Ari Palomäki

**Affiliations:** School of Medicine, University of Tampere, Tampere, Finland; Department of Obstetrics and Gynaecology, Tampere University Hospital, Tampere, Finland; Department of Obstetrics and Gynaecology, Kanta-Häme Central Hospital, Hämeenlinna, Finland; Department of Emergency Medicine, Kanta-Häme Central Hospital, Hämeenlinna, Finland

**Keywords:** Gestational diabetes mellitus, Metabolic syndrome, Body mass index, Body weight excess, Cardiovascular risk factors

## Abstract

**Background:**

Women with gestational diabetes mellitus (GDM) are at an increased risk of developing metabolic syndrome (MetS) after delivery. Recently, the prevalence of both GDM and MetS has increased worldwide, in parallel with obesity. We investigated whether the presentation of MetS and its clinical features among women with previous GDM differs from that among those with normal glucose tolerance during pregnancy, and whether excess body weight affects the results.

**Methods:**

This hospital-based study of two cohorts was performed in Kanta-Häme Central Hospital, Finland. 120 women with a history of GDM and 120 women with a history of normal glucose metabolism during pregnancy, all aged between 25 and 46 were enrolled. They all underwent physical examination and had baseline blood samples taken. All 240 women were also included in subgroup analyses to study the effect of excess body weight on the results.

**Results:**

Although the groups did not differ in body mass index (BMI; p = 0.069), the risk of developing MetS after pregnancy complicated by GDM was significantly higher than after normal pregnancy, 19 vs. 8 cases (p  =  0.039). Fasting glucose (p < 0.001) and triglyceride levels (p < 0.001) were significantly higher in women affected. In subgroup analysis, cardiovascular risk factors were more common in participants with high BMI than in those with previous gestational diabetes.

**Conclusions:**

The risk of MetS was 2.4-fold higher after GDM than after normal pregnancy. Cardiovascular risk factors were more common in participants with high BMI than in those with previous GDM. Multivariate analysis supported the main findings. Weight control is important in preventing MetS after delivery.

## Introduction

The prevalence of gestational diabetes mellitus (GDM) has increased globally in recent decades along with increasing rates of obesity and inactive lifestyles [1,2]. In Finland, GDM affected 15.0% of pregnancies in 2013 [1]. Glucose intolerance normalizes after delivery in most cases [3,4], but women with a history of GDM have at least a sevenfold risk of developing type 2 diabetes in the future [5]. Affected women are also at an increased risk of developing cardiovascular disease or metabolic syndrome (MetS) years after the pregnancy [6-9].

Metabolic syndrome is an international health problem considered to be the result of concomitant accumulation of abdominal obesity, hypertension, dyslipidaemia and abnormal glucose tolerance or diabetes [10]. In recent decades, the prevalence of MetS has rapidly increased in parallel with sedentary lifestyles [6], leading to major healthcare costs. The chance of developing cardiovascular disease is six to eight times higher and that of mortality related to cardiovascular disease two to three times higher among the MetS population than among healthy controls [11-14].

Gestational diabetes mellitus shares common features with MetS, including dyslipidaemia, insulin resistance and endothelial dysfunction [15-19]. Several studies have revealed an increased risk of MetS in association with a history of GDM [7,20,21]. For example, a Danish study demonstrated that the prevalence of MetS in women with a history of GDM was threefold higher than in the general age-matched population [7]. However, other studies have shown contrasting results, with no association between GDM and MetS [22,23].

Women’s health after GDM has been widely studied. However, the effect of an overweight condition on health after GDM or after normal pregnancy is less well known. The aim of our hospital-based study of two age-matched cohorts was to reveal whether or not the presentation of MetS and its individual variables among women with previous GDM differs from those with normal glucose metabolism a few years after delivery. In this first study of the Hämeenlinna GDM Research Programme, we also wanted to investigate if there is a difference in clinical features between the groups and whether excess body weight affects the results.

## Methods

We investigated a total of 120 parturients from our area aged 25 to 46 years and with a history of GDM during the index pregnancy and we compared them with 120 age-matched women with normal glucose metabolism during pregnancy. Power analyses were conducted to estimate the required number of participants. Concerning continuous variables, we worked on a difference of 10% with a standard deviation of 25% (Cohen’s d = 0.40). Regarding the presentation of MetS the expected proportions were 10% and 25%. When the significance level was set at 5% and power at 80%, the estimated numbers of participants as regards continuous and categorial variables were 99 and 100 in both groups, respectively. In Kanta-Häme Central Hospital, Finland, there are approximately 1700 deliveries annually. The electronic database of the hospital was used to pick up the cases and controls. Both recruitment and examinations were carried out between August 2011 and July 2014.

Inclusion criteria were as follows:

- Index pregnancy and delivery 2–6 years before participating in the study
- GDM group: GDM defined as a pathological value in the 75-g oral glucose tolerance test (OGTT) during the pregnancy; venous plasma glucose ≥  5.3 mmol/L when fasting, ≥ 10.0 mmol/L at 1 hour or ≥  8.6 mmol/L at 2 hours. The diagnostic criteria of GDM were the same as in current Finnish guidelines [24].
- Control group: normal OGTT results during the pregnancy and birth weight of the newborn <  4.5 kg

Exclusion criteria were as follows:

- Multiple pregnancy
- Suspected or verified endocrine or malignant disease
- Treatment of or known clinical history of psychiatric illness
- Substance abuse
- GDM group: diagnosed type 1 or 2 diabetes before the index pregnancy
- Control group: GDM in earlier pregnancy

Resting blood pressure and heart rate, weight (kg), height (cm) and waist circumference (cm) of the participants were measured. Body mass index (BMI) was calculated as weight in kilograms divided by height in meters squared (kg/m^2^). Metabolic syndrome was defined according to the National Cholesterol Education Program (NCEP Adult Treatment Panel III) as the presence of at least three of the following five criteria [10]:

- waist circumference > 88 cm
- serum triglycerides ≥ 1.7 mmol/L
- serum high-density lipoprotein (HDL) cholesterol level < 1.3 mmol/L
- blood pressure ≥ 130/85 mmHg
- plasma glucose level ≥ 6.1 mmol/L or diabetes mellitus

Further, we interviewed the participants as regards their medical histories and lifestyle habits. Initially successful weight loss followed by weight regain (so called “yo-yo” dieting or weight cycling) is associated with body weight excess and abdominal fat accumulation [25]. To analyse “yo-yo” dieting, we estimated total lifetime weight loss by adding together kilograms lost during every previous intentional weight-loss period. Lifetime tobacco exposure was calculated as pack-years by multiplying smoking years with average packs smoked daily [26]. One pack-year is defined as twenty cigarettes smoked every day for one year.

The primary outcome was to define the prevalence of MetS and its different variables in the GDM and control groups. We also wanted to see if there were differences in medical history, lifestyle habits, pregnancy outcomes or clinical characteristics between the groups. The secondary aim was to investigate the influence of excess body weight on these results.

Every participant was given both oral and written information on the study before she signed an informed consent document. The study protocol was approved by the Ethics Committee of Kanta-Häme Hospital District and the study followed the ethical principles outlined in the Declaration of Helsinki [27].

Basic blood count and serum levels of creatinine, alanine transaminase (ALAT), fasting glucose, total cholesterol, HDL cholesterol, low-density-lipoprotein (LDL) cholesterol and triglycerides, and the urinary albumin to creatinine ratio, as well as fibrinogen, were analysed according to validated methods after at least 12 hours of fasting. Direct analyses of total cholesterol, HDL cholesterol, LDL cholesterol and triglycerides were carried out by using commercial reagents from Beckman Coulter (Brea, CA, USA). Analyses of ALAT (IFCC method), creatinine (Jaffé method) and plasma glucose (hexokinase method) were carried out by using commercial reagents from Beckman Coulter, with an Olympus AU640 analyser and analyses of fibrinogen (Clauss method) by using Siemens BCS XP equipment.

### Statistical analyses

Statistics were analysed by using IBM® SPSS® Statistics Version 22 software (copyright 2013). Variables were tested for normality by way of Shapiro–Wilk or Kolmogorov–Smirnov tests, as appropriate. Data are presented as mean ± standard deviation (SD) if not mentioned otherwise. Differences in continuous variables between GDM participants and controls were studied by using Student's *t*-test in cases of normality and by the Mann–Whitney *U*-test in cases of non-normality. Categorial data are presented as percentages and were compared by using the chi-square test. All 240 women were also included in subgroup analyses to study the effect of excess body weight on the results. For these analyses, we divided the whole study group into two halves according to BMI, using a cut-off point of 27 kg/m^2^. According to the FINRISK 2012 Study our BMI cut-off of 27 kg/m^2^ relatively well represents average BMI among Finnish women [28]. Medicines agencies also define the cut-off point of overweight as a BMI of 27 kg/m^2^ [29]. There were 122 women in the “obese” group (BMI ≥ 27); 65 GDM and 57 control participants. The “non-obese” group (BMI < 27; n = 118) consisted of 55 GDM and 63 control participants. The clinical characteristics of these four subgroups were studied by way of one-way ANOVA in cases of normality and by using the Kruskal–Wallis test in cases of non-normality. Post hoc analyses were performed, when appropriate. Logistic regression analysis was carried out to identify predictors as regards the presentation of MetS. First, univariate analysis was carried out. The set of independent variables tested included previous GDM, maternal age, BMI, family history of diabetes mellitus, pack-years of smoking, total lifetime weight loss, method of treatment among GDM cases, birth weight of the newborn, time from delivery to the present study and serum concentration of total cholesterol. The significant independent variables were then entered into multivariate analysis. The results are expressed as odds ratios (ORs) and 95% confidence intervals (CIs). A two-tailed probability value of < 0.05 was considered significant.

## Results

Basic information on the index pregnancy in the GDM and control groups is shown in Table 1. All GDM participants and controls underwent a 75-g OGTT during the index pregnancy. A total of 25 GDM participants had medication during their pregnancies (insulin, n  =  24; metformin, n  =  1), while the other mothers in the GDM group had only dietary therapy. Twenty-three of the 120 women were primiparous in both groups. Nearly a third (29.9%, n  =  29/97) of the multiparous GDM participants had already experienced GDM in an earlier pregnancy. Accumulation of gestational hypertension and pre-eclampsia was more common in diabetic pregnancies (p = 0.038). There was more glucosuria and proteinuria in pregnancies affected by GDM, as shown in Table 1.

**Table 1:** **Characteristics of the index pregnancy in the GDM and control groups**

	**GDM**	**Control**	***p*** **value**
	**(n = 120)**	**(n = 120)**	
**75-g OGTT**			
- 0 h, mmol/L	5.4 ± 0.5	4.7 ± 0.3	< 0.001
- 1 h, mmol/L	9.5 ± 2.3	7.1 ± 1.4	< 0.001
- 2 h, mmol/L	7.7 ± 2.0	5.8 ± 1.0	< 0.001
**Pregnancy disorders**			
- Gestational hypertension, n (%)	12 (10%)	6 (5%)	NS
- Pre-eclampsia, n (%)	7 (5.8%)	2 (1.7%)	NS
- Glucosuria, n (%)	25 (20.8%)	4 (3.3%)	< 0.001
- Proteinuria, n (%)	19 (15.8%)	7 (5.8%)	0.021
**Induction of delivery, n (%)**	42 (35.0%)	26 (21.7%)	0.031
**Caesarean section, n (%)**	29 (24.2%)	21 (17.5%)	NS
**Perinatal outcome**			
- Gestational age, days	277.1 ± 9.5	278.8 ± 10.4	NS
- Birth weight of the child, g	3633 ± 519	3540 ± 471	NS
- Apgar score at one minute	8.6 ± 1.2	8.7 ± 1.4	NS
- Apgar score at five minute	9.3 ± 0.8	9.3 ± 0.8	NS
- Umbilical blood arterial pH	7.29 ± 0.1	7.28 ± 0.1	NS (0.054)
- Umbilical blood venous pH	7.35 ± 0.1	7.35 ± 0.1	NS

Data are presented as mean ± SD if not mentioned otherwise.

OGTT: Oral glucose tolerance test.

**Figure 1:**
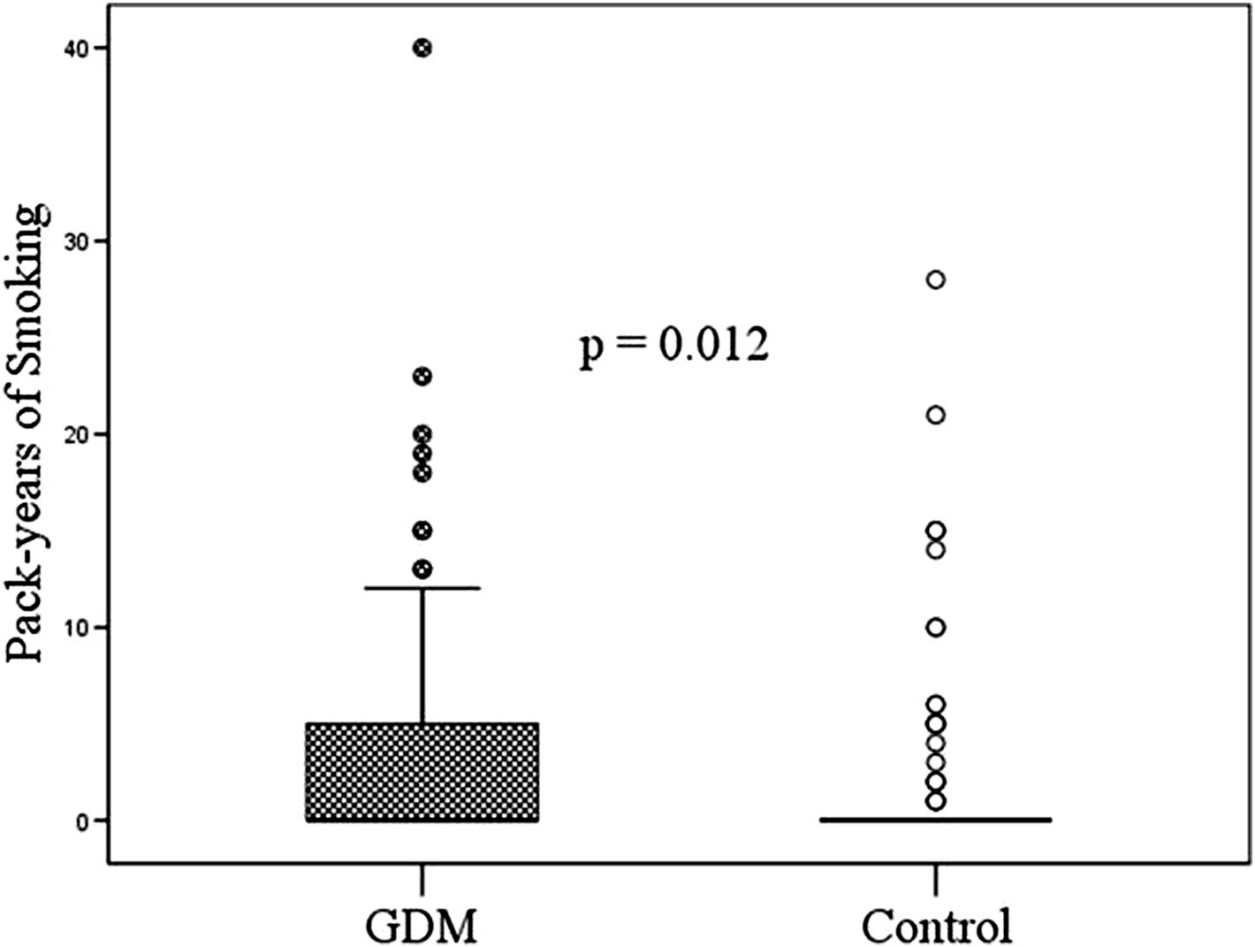
Pack-years of smoking in the GDM and control groups. Pack-years of smoking differed significantly (p = 0.012) between women with a previous history of GDM vs. women unaffected. The median in both groups was zero, because the majority were non-smokers. The mean (±SD) number of pack-years in the GDM group was 3.1 (±6.1) and in the control group, 1.6 (±4.4).

The average time to follow-up was 3.7 years in both study groups. Clinical characteristics in women with and without previous GDM are shown in Table 2. According to our study interview data there were more current or former smokers in the GDM group than in the control group, and also the pack-years of smoking differed significantly (Figure 1). The groups did not differ in physical activity, alcohol intake or lifetime weight loss. The GDM group used less margarine weekly than the control group (n  =  64 vs. 81; p = 0.034), but on the other hand the groups did not differ in weekly use of butter (n  =  69 vs. 66). The GDM participants also consumed fewer sweets and sweet baked goods weekly (n  =  95 vs. 111; p = 0.005) than the controls. Otherwise, we found no other differences in basic nutrition habits between the groups.

**Table 2:** **Clinical characteristics in the GDM and control groups**

	**GDM**	**Control**	***p*** **value**
	**(n = 120)**	**(n = 120)**	
**Age at follow-up, years**	35.8 ± 4.4	35.9 ± 4.6	NS
**Family history of**			
- Coronary heart disease, n (%)	20 (16.7%)	23 (19.2%)	NS
- Cerebrovascular disease, n (%)	15 (12.5%)	5 (4.2%)	0.033
- Diabetes mellitus, n (%)	32 (26.7%)	27 (22.5%)	NS
**Diagnosed disorder, n (%)**	52 (43.3%)	45 (37.5%)	NS
- Hypertension, n (%)	3 (2.5%)	5 (4.2%)	NS
- Type 1 diabetes mellitus, n (%)	2 (1.7%)	0 (0%)	NS
- Type 2 diabetes mellitus, n (%)	1 (0.8%)	0 (0%)	NS
- Polycystic ovary syndrome, n (%)	8 (6.7%)	1 (0.8%)	0.036
**Permanent medication for any chronic disease, n (%)**	43 (35.8%)	35 (29.2%)	NS
**Contraception, n (%)**	99 (82.5%)	92 (76.7%)	NS
**Smoking status**			0.018
- Current, n (%)	24 (20.0%)	12 (10%)	
- Former, n (%)	45 (37.5%)	37 (30.8%)	
- Never, n (%)	51 (42.5%)	71 (59.2%)	
**BMI, kg/m2**	28.3 ± 5.0	27.5 ± 5.4	NS (0.069)
**Waist circumference, cm**	96.8 ± 13.0	92.5 ± 12.6	0.009
**Systolic blood pressure, mmHg**	122.4 ± 12.5	119.0 ± 11.5	0.034
**Diastolic blood pressure, mmHg**	73.5 ± 9.0	71.8 ± 8.7	NS
**Heart rate, beats per minute**	65.9 ± 9.1	63.8 ± 9.6	0.017
**MetS, n (%)**	19 (15.8%)	8 (6.7%)	0.039
- Waist circumference > 88 cm, n (%)	89 (74.2%)	73 (60.8%)	0.038
- Blood pressure ≥ 130/85 mmHg, n (%)	35 (29.2%)	25 (20.8%)	NS
- HDL cholesterol < 1.30 mmol/L, n (%)	23 (19.2%)	22 (18.3%)	NS
- Triglycerides ≥ 1.7 mmol/L, n (%)	12 (10.0%)	5 (4.2%)	NS (0.084)
- Glucose ≥ 6.1 mmol/L or diabetes, n (%)	18 (15.0%)	4 (3.3%)	0.002

Data are presented as mean ± SD if not mentioned otherwise. Metabolic syndrome and separate variables defined by NCEP.

Despite a current Finnish guideline recommending OGTT screening six to twelve weeks after delivery in cases of medicated GDM during pregnancy, and one year after delivery in diet-treated GDM during pregnancy [24], only 41 of the 120 women (34.2%) with a history of GDM had an OGTT after delivery. Of these, 39.0% (16/41) showed glucose intolerance as follows: 17.1% (7/41) had impaired fasting glucose (IFG), 14.6% (6/41) had impaired glucose tolerance (IGT) and 7.3% (3/41) had diabetes. The results of OGTTs were normal in 25 of the 41 cases.

Clinical chemical data concerning the women with and without previous GDM are presented in Table 3. Between the groups, there were significant differences in serum concentrations of fasting glucose and triglycerides, both of them variables of MetS. When GDM participants with medication (n = 25) were compared with those with dietary therapy (n = 95) during the index pregnancy, we noticed a significant difference only in fasting glucose (6.0 ± 1.0 vs. 5.5 ± 0.4 mmol/L; p = 0.003). As shown in Table 2, the women in the GDM group met the criteria of MetS 2.4-fold more often than did the controls. The numbers of participants with separate variables of metabolic syndrome defined by NCEP are also shown in Table 2.

**Table 3:** **Laboratory characteristics of participants with GDM vs. controls**

	**GDM**	**Control**	***p*** **value**
	**(n = 119)**	**(n = 120)**	
**Leucocytes, 109/L**	5.8 ± 1.6	5.2 ± 1.4	0.008
**Haemoglobin, g/L**	133.2 ± 9.3	128.6 ± 12.9	0.001
**Platelets, 109/L**	241.9 ± 58.2	244.0 ± 52.5	NS
**ALAT, U/L**	22.8 ± 17.4	19.7 ± 10.5	NS
**Creatinine, umol/L**	66.6 ± 7.7	64.5 ± 7.8	0.048
**U-AlbCre, mg/mmol**	0.67 ± 0.5	0.57 ± 0.3	NS (0.070)
**Fibrinogen, g/L**	3.4 ± 0.9	3.2 ± 1.0	NS (0.096)
**Fasting glucose, mmol/L**	5.6 ± 0.6	5.3 ± 0.3	< 0.001
**Total cholesterol, mmol/L**	4.7 ± 0.9	4.6 ± 0.8	NS
**HDL cholesterol, mmol/L**	1.5 ± 0.3	1.6 ± 0.3	NS
**LDL cholesterol, mmol/L**	3.0 ± 0.7	2.8 ± 0.6	NS
**Triglycerides, mmol/L**	1.1 ± 0.6	0.9 ± 0.4	< 0.001

Data are presented as mean ± SD.

U-AlbCre: urinary albumin to creatinine ratio, ALAT: alanine transaminase.

**Figure 2:**
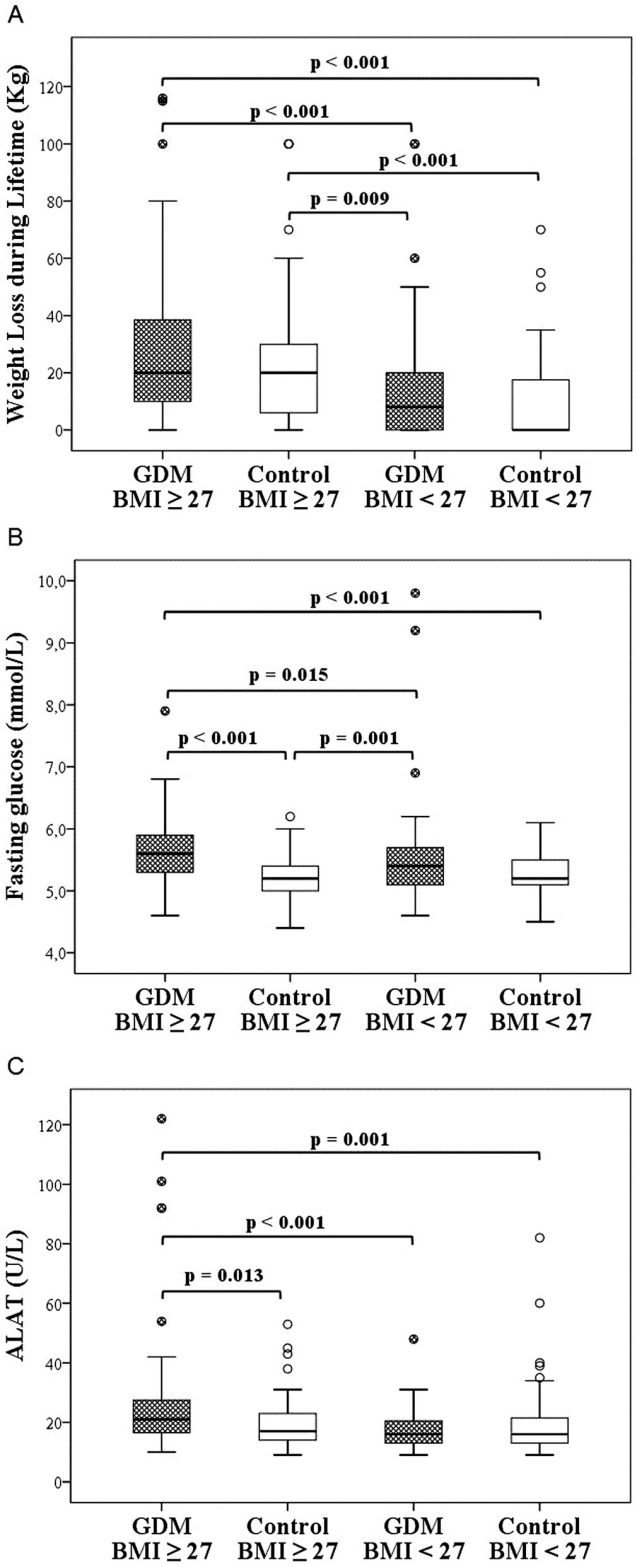
Lifetime weight loss, fasting glucose and alanine transaminase in the subgroups. **A**: Median (minimum, maximum) lifetime weight loss among obese (BMI ≥ 27) GDM women was 20 (0, 116) kg, among obese control women 20 (0, 100) kg, among non-obese GDM women 8 (0, 100) kg and among non-obese control women 0 (0, 70) kg. **B**: Median (minimum, maximum) fasting glucose levels among obese GDM women 5.6 (4.6, 7.9) mmol/L, among obese control women 5.2 (4.4, 6.2) mmol/L, among non-obese GDM women 5.4 (4.6, 9.8) mmol/L and among non-obese control women 5.2 (4.5, 6.1) mmol/L. **C**: Median (minimum, maximum) alanine transaminase levels among obese GDM women 21 (10, 122) U/L, among obese control women 17 (9, 53) U/L, among non-obese GDM women 16 (9, 48) U/L and among non-obese control women 16 (9, 82).

**Figure 3:**
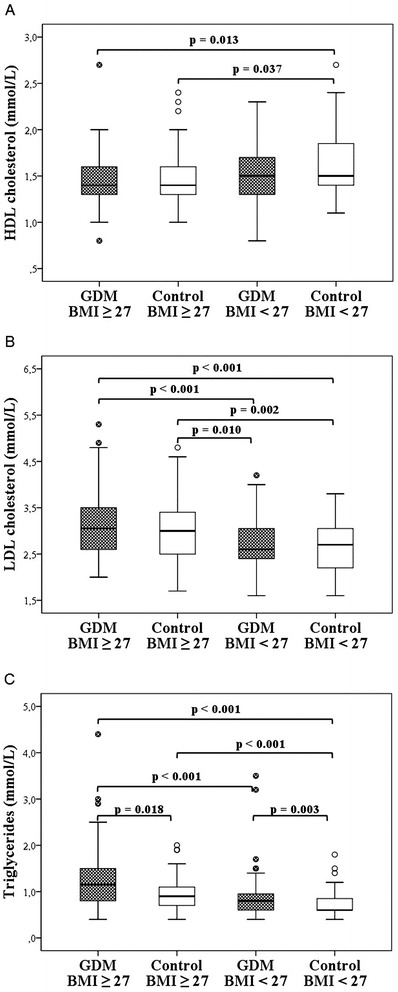
HDL cholesterol, LDL cholesterol and triglyceride levels in the subgroups. **A**: The median (minimum, maximum) HDL cholesterol level among obese (BMI ≥ 27) GDM women was 1.4 (0.8, 2.7) mmol/L, among obese control women 1.4 (1.0, 2.4) mmol/L, among non-obese GDM women 1.5 (0.8, 2.3) mmol/L and among non-obese control women 1.5 (1.1, 2.7) mmol/L. **B**: The median (minimum, maximum) LDL cholesterol level among obese GDM women was 3.1 (2.0, 5.3) mmol/L, among obese control women 3.0 (1.7, 4.8) mmol/L, among non-obese GDM women 2.6 (1.6, 4.2) mmol/L and among non-obese control women 2.7 (1.6, 3.8) mmol/L. **C**: The median (minimum, maximum) triglyceride level among obese GDM women was 1.2 (0.4, 4.4) mmol/L, among obese control women 0.9 (0.5, 2.0) mmol/L, among non-obese GDM women 0.8 (0.4, 3.5) mmol/L and among non-obese control women 0.6 (0.4, 1.8) mmol/L.

In subgroup analyses, MetS affected participants in obese subgroups more often than in non-obese subgroups, as shown in Table 4. These four subgroups, obese GDM cases and their controls, and non-obese GDM cases and their controls, did not differ significantly in family history of cardio- or cerebrovascular diseases, medical history, medication, contraception, physical activity or alcohol consumption. Pack-years of smoking among non-obese GDM women were 2.7 (±3.5), among obese GDM women 4.7 (±7.5), among non-obese control women 1.6 (±3.5) and among obese control women 3.3 (±5.5) (p = 0.058). The subgroups did not differ significantly in perinatal outcomes either. There was a major difference in lifetime weight loss (Figure 2A), both obese GDM and obese control women having lost more weight than non-obese GDM and control women. There were differences in most of the basic clinical characteristics between these four subgroups, particularly between non-obese and obese subgroups, as demonstrated in Figures 2B, C and 3A–C, and Table 4.

**Table 4:** **Clinical characteristics of non-obese GDM cases and their controls, and obese GDM cases and their controls**

	**GDM cases**		**Controls**		**Overall**
	**BMI ≥ 27**	**BMI < 27**	**BMI ≥ 27**	**BMI < 27**	***p*** **value**
	**(n = 65)**	**(n = 55)**	**(n = 57)**	**(n = 63)**	
Systolic blood pressure, mmHg*	126.6 ± 12.3	117.7 ± 11.2	122.8 ± 12.4	116.1 ± 9.1	< 0.001
Diastolic blood pressure, mmHg*	76.1 ± 9.6	70.5 ± 9.6	74.6 ± 8.1	69.1 ± 8.5	< 0.001
Mean peripheral pressure, mmHg*	94.0 ± 10.7	87.0 ± 8.4	91.5 ± 9.3	85.3 ± 8.8	< 0.001
**Heart rate, beats per minute**	66.6 ± 8.9	65.2 ± 9.3	65.2 ± 9.0	62.6 ± 10.1	NS
**MetS, n (%)**	15 (23.1 %)	4 (3.3 %)	8 (14.0 %)	0 (0 %)	< 0.001
- Waist circumference > 88 cm, n (%)	62 (95.4 %)	27 (49.1 %)	53 (93.0 %)	20 (31.7 %)	< 0.001
- Blood pressure ≥ 130/85 mmHg, n (%)	27 (41.5 %)	8 (14.5 %)	19 (33.3 %)	6 (9.5 %)	< 0.001
- HDL cholesterol < 1.30 mmol/L, n (%)	14 (21.5 %)	9 (16.4 %)	14 (24.6 %)	8 (12.7 %)	NS
- Triglycerides ≥ 1.7 mmol/L, n (%)	9 (13.8 %)	3 (5.5 %)	4 (7.0 %)	1 (1.6 %)	NS (0.050)
- Glucose ≥ 6.1 mmol/L or diabetes, n (%)	11 (16.9 %)	7 (12.7%)	1 (1.8 %)	3 (4.8 %)	0.012

Metabolic syndrome and separate variables defined by NCEP.

Data are presented as mean ± SD if not mentioned otherwise.

*Differences between non-obese GDM cases and their controls, and obese GDM cases and their controls were non-significant; differences in other subgroup comparisons were significant.

In univariate logistic regression analysis, previous GDM (OR 2.63, 95% CI 1.11–6.28; p = 0.029), higher BMI values (OR 1.24, 95% CI 1.14–1.35; p < 0.001), greater lifetime weight loss (OR 1.02, 95% CI 1.00–1.03; p = 0.013) and higher levels of total cholesterol (OR 1.98, 95% CI 1.26–3.10; p = 0.003) were associated with an increased risk of MetS. Multivariate analysis also showed that previous GDM (OR 2.83, 95% CI 1.05–7.63; p = 0.040), higher BMI values (OR 1.24, 95% CI 1.13–1.36; p < 0.001) and higher serum concentrations of total cholesterol (OR 1.68, 95% CI 1.01–2.79; p = 0.046) seemed to predict the presentation of MetS. No other associations were found in logistic regression analyses.

## Discussion

The main finding in our study was that the risk of developing MetS after GDM was 2.4-fold greater than after normal pregnancy. However, cardiovascular risk factors such as increased LDL cholesterol and triglyceride levels as well as decreased HDL cholesterol concentrations were more common in participants with high BMI than in those with previous GDM.

A systematic review conducted in 2014 demonstrated that women who have had GDM have a nearly fourfold increased risk of developing MetS in the future than those who have had a normal pregnancy. However, there are some factors that may modify the risk of developing MetS after GDM. For example, ethnicity may significantly affect MetS susceptibility. BMI is also an important confounder in the overall MetS risk estimate. When MetS after GDM was grouped by BMI, the odds ratio was 2.53 according to recent meta-analyses [6]. In our study, both the participants and the controls were of Caucasian origin, and there was no significant difference between the groups in BMI or body weight. Our results are in accordance with results reported earlier [6].

The results of previous studies indicate that there is a relationship among the risk gene variants as regards both GDM and MetS [30-32]. Possibly, genetic factors also protect obese control women against insulin resistance and, on the other hand, expose non-obese or even lean GDM women to glucose intolerance during pregnancy. At the same time, non-obese GDM women seem to have a better cardiovascular profile a few years after their index pregnancies than both obese groups. Cross-sectional analysis of different variables does not foretell the prognosis of women in the future. According to our results, obesity seems to represent a greater risk of MetS and presentation of cardiovascular risk variables than previous GDM, at least after a few years of delivery. The results of multivariate analysis supported the main findings.

A strength of our study is that all participants had undergone OGTT screening during the index pregnancy. In Finland, GDM screening via 75-g OGTTs is offered to all pregnant women at risk of GDM. Current care guidelines in Finland do not recommend OGTT screening for low-risk women – primiparous women < 25 years old, BMI ≤ 25 kg/m^2^, and no family history of DM, or multiparous women < 40 years old, no GDM in previous pregnancy or pregnancies, and BMI ≤ 25 kg/m^2^ before the current pregnancy [24].

OGTT screening has been carried out in 51.5% of pregnancies during the past five years in our area. We wanted to be sure that the controls really were unaffected as regards glucose intolerance and had undergone OGTTs during their index pregnancies. This situation could reflect a hidden weakness of our study, since maybe the best controls, being part of the 48.5% low-risk parturients who did not undergo OGTT screening during pregnancy, were excluded from the study. Another ambiguous matter was the BMI cut-off point of 27 kg/m^2^, because obesity is commonly classified as BMI of ≥ 30 kg/m^2^ [33]. In our subgroup analysis, we used BMI to divide our study group into two halves, intending to reveal the effect of excess body weight on cardiovascular risk factors. According to the FINRISK 2012 Study, mean BMI among women aged 25–74 years is 26.8 kg/m^2^ in Finland [28], so actually our cut-off point of BMI fairly well represents average BMI among Finnish women. Medicines agencies in Europe and in the USA define the cut-off point of overweight as a BMI of 27 kg/m^2^. Arguments for this definition have been discussed in detail earlier [29].

Women who have had GDM are advised to have glucose tolerance assessed postpartum [24,34]. The low rate of attendance at follow-up suggests that many healthcare providers may not recognize GDM as an initial warning sign of predisposition to MetS. In Finland, there is no consensus of opinion regarding how to monitor obese women after normal pregnancy, but according to our results, we suggest that unaffected obese women should undergo screening for at least cardiovascular risk factors after delivery. Paying attention to patients with pathological OGTT results as well as an overweight condition during and after pregnancy helps healthcare professionals to identify women who may be at risk of developing MetS.

## Conclusions

In conclusion, the risk of metabolic syndrome was 2.4 times higher after GDM compared with normoglycaemic pregnancy, but the risk factors of coronary heart disease were even more evident in women with excess body weight. Women with previous GDM, particularly obese ones, and also unaffected obese women should not miss the opportunity to prevent future metabolic disease.

## Abbreviations

ALAT: Alanine transaminase
BMI: Body mass index
GDM: Gestational diabetes mellitus
HDL: High density lipoprotein
LDL: Low density lipoprotein
MetS: Metabolic syndrome
NCEP: National cholesterol education program
OGTT: Oral glucose tolerance test
OR: Odds ratio
CI: Confidence interval
